# Variability in Global Prevalence of Interstitial Lung Disease

**DOI:** 10.3389/fmed.2021.751181

**Published:** 2021-11-04

**Authors:** Bhavika Kaul, Vincent Cottin, Harold R. Collard, Claudia Valenzuela

**Affiliations:** ^1^Department of Medicine, University of California, San Francisco, San Francisco, CA, United States; ^2^Department of Respiratory Medicine, National Coordinating Reference Center for Rare Pulmonary Diseases, Louis Pradel Hospital, Hospices Civils de Lyon, Lyon, France; ^3^IVPC, INRAE, Claude Bernard University Lyon 1, Member of ERN-LUNG, Lyon, France; ^4^Interstitial Lung Disease Unit Pulmonology Department, Hospital Universitario de La Princesa, Universidad Autonoma de Madrid, Madrid, Spain

**Keywords:** interstitial lung disease, epidemiology—descriptive, global epidemiology, idiopathic pulmonary fibrosis, mortality

## Abstract

There are limited epidemiologic studies describing the global burden and geographic heterogeneity of interstitial lung disease (ILD) subtypes. We found that among seventeen methodologically heterogenous studies that examined the incidence, prevalence and relative frequencies of ILDs, the incidence of ILD ranged from 1 to 31.5 per 100,000 person-years and prevalence ranged from 6.3 to 71 per 100,000 people. In North America and Europe, idiopathic pulmonary fibrosis and sarcoidosis were the most prevalent ILDs while the relative frequency of hypersensitivity pneumonitis was higher in Asia, particularly in India (10.7–47.3%) and Pakistan (12.6%). The relative frequency of connective tissue disease ILD demonstrated the greatest geographic variability, ranging from 7.5% of cases in Belgium to 33.3% of cases in Canada and 34.8% of cases in Saudi Arabia. These differences may represent true differences based on underlying characteristics of the source populations or methodological differences in disease classification and patient recruitment (registry vs. population-based cohorts). There are three areas where we feel addition work is needed to better understand the global burden of ILD. First, a standard ontology with diagnostic confidence thresholds for comparative epidemiology studies of ILD is needed. Second, more globally representative data should be published in English language journals as current literature has largely focused on Europe and North America with little data from South America, Africa and Asia. Third, the inclusion of community-based cohorts that leverage the strength of large databases can help better estimate population burden of disease. These large, community-based longitudinal cohorts would also allow for tracking of global trends and be a valuable resource for collective study. We believe the ILD research community should organize to define a shared ontology for disease classification and commit to conducting global claims and electronic health record based epidemiologic studies in a standardized fashion. Aggregating and sharing this type of data would provide a unique opportunity for international collaboration as our understanding of ILD continues to grow and evolve. Better understanding the geographic and temporal patterns of disease prevalence and identifying clusters of ILD subtypes will facilitate improved understanding of emerging risk factors and help identify targets for future intervention.

## Introduction

Interstitial lung disease (ILD) describes a heterogenous group of disorders that are subclassified based on similar radiographic or pathologic manifestations. Although several classification schemes exist, generally, ILDs can be subcategorized into: (1) those that occur secondary to a known cause such as a culprit drug or connective tissues disease, (2) idiopathic interstitial pneumonias of which idiopathic pulmonary fibrosis (IPF) is the most common, (3) granulomatous parenchymal lung disease such as sarcoidosis or hypersensitivity pneumonitis, (4) occupational pneumoconiosis, and (5) other rarer forms of diffuse parenchymal lung disease (1, 2).

Prior literature describing the epidemiology of ILDs has utilized national registries, health insurance claims, and social security databases to quantify incidence and prevalence, identify risk factors, and describe disease behavior (clinical presentation, natural history, and outcomes) (3, 4), with a growing body of literature focused on the epidemiology of IPF. Very few studies have examined the global burden of ILD or described the between country variability in disease prevalence and subtype. Better quantifying the geographic burden of ILD and understanding the regional variability can lend insight into new risk factors and identify targets for prevention and intervention. It can also help healthcare systems make informed decisions on how best to allocate resources to meet local needs, which is of particular importance in an era of emerging ILD therapies. The objective of this narrative review is to describe what is known from the English language literature about the geographic variability in ILD prevalence and subtype, discuss potential reasons for the observed heterogeneity, and define current knowledge gaps for future investigation.

We queried the PubMed database to identify relevant studies describing ILD epidemiology. Combination of search terms “epidemiology,” “interstitial lung disease,” “pulmonary fibrosis,” and “prevalence” were used to identify English language studies in humans that had the key search terms in their title or abstract. All abstracts were reviewed for relevance. We excluded studies that focused on a single ILD (ex. IPF only) or were intentionally enriched for certain types of ILD as the goal of this review was to describe the comparative frequency of ILD subtypes. References of key articles were reviewed to supplement the electronic search. A total of 17 studies that described incidence, prevalence and relative frequency of ILD subtypes were identified.

## Comparative Epidemiology of Interstitial Lung Disease

### North America

**Figure 1:**
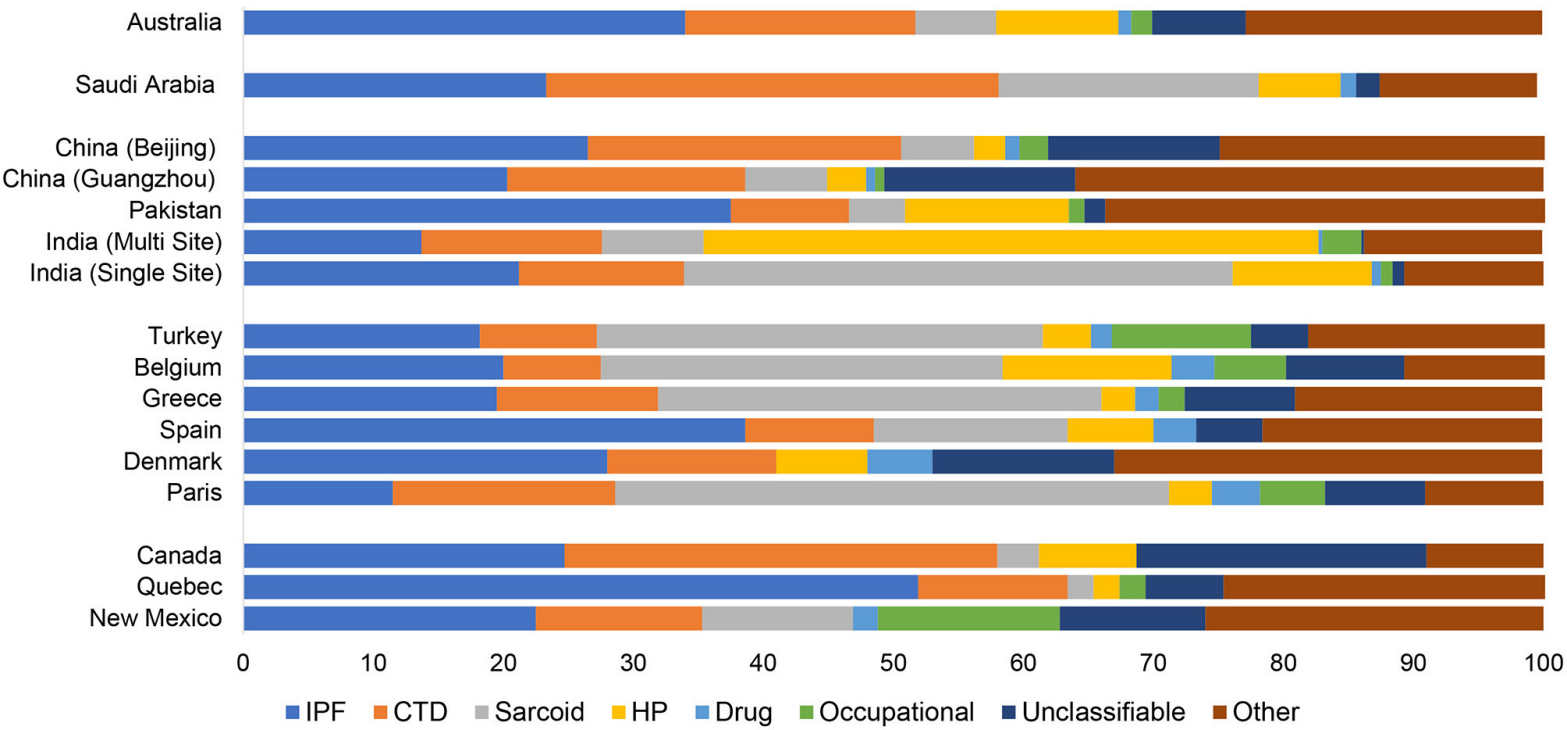
Relative frequency of interstitial lung disease subtype by geography. IPF, idiopathic pulmonary fibrosis; CTD, connective tissue disease; HP, hypersensitivity pneumonitis.

One of the first epidemiological studies to evaluate the comparative frequencies of ILDs examined the population burden of disease in Bernalillo County, New Mexico between 1988 and 1990 (5). Patients with ILD were identified through a combination of physician referrals, hospital discharge diagnosis, histopathology reports, and death certificates. Electronic health records were reviewed for diagnostic ascertainment. The median age was 69 years and 52.5% of the cohort was male. The incidence of ILD was 26.1 per 100,000 person-years among women and 31.5 per 100,000 person-years among men (Table 1). The prevalence of ILD was 67.2 cases per 100,000 among women and 80.9 cases per 100,000 among men. IPF was the most common ILD, representing 22.5% of prevalent cases, followed by occupational lung disease (14%), connective tissue disease (CTD) ILD (12.8%), and sarcoidosis (11.6%) (Table 2, Figure 1). The overall prevalence of ILD was 20% higher in males than females, which was driven in part by a higher prevalence of occupational lung disease among men (20.8 per 100,000) compared to women (0.6 per 100,000). Mining is a major industry in New Mexico, which the authors hypothesized likely contributed to the higher prevalence of pneumoconiosis in the male population.

**Table 1:** Incidence and prevalence of interstitial lung disease subtypes.

		**Time Period**	**ILD** **(All Subtypes)**	**IPF**	**CTD**	**Sarcoid**	**HP**	**Drug**	**Occupational**	**Unclassifiable**
**North America**										
New Mexico, USA	Incidence	1988–1990	Male 31.5 Female 26.1	Male 10.7 Female 7.4	Male 2.1 Female 3.0	Male 0.9 Female 3.6	–	Male 1.8 Female 1.1	Male 6.2 Female 0.8	–
New Mexico, USA	Prevalence	1988–1990	Male 80.9 Female 67.2	Male 20.2 Female 13.2	Male 7.1 Female 11.6	Male 8.3 Female 8.8	–	Male 1.2 Female 2.2	Male 20.8 Female 0.6	–
**Europe**										
Flanders (Belgium)	Incidence	1992–1996	1.0	0.22	0.07	0.26	0.12	0.05	0.07	0.10
Flanders (Belgium)	Prevalence	1992–1996	6.27	1.25	0.47	1.94	0.81	0.21	0.35	0.57
Greece	Incidence	2004	4.63	0.93	0.54	1.07	0.13	0.07	0.14	0.71
Greece	Prevalence	2004	17.3	3.38	2.14	5.89	0.45	0.30	0.36	1.46
Denmark	Incidence	2003–2009	4.1	1.3	–	–	–	–	–	–
Paris, France	Incidence	2012	18.3	2.8	3.3	4.9	0.9	1.2	0.8	1.8
Paris, France	Prevalence	2012	71.0	8.2	12.1	30.2	2.3	2.6	3.5	5.0
Turkey	Incidence	2007–2009	25.8	–	–	4.0	–	–	–	–

*Incidence and prevalence defined as cases per 100,000*.

*ILD, interstitial lung disease; IPF, idiopathic pulmonary fibrosis; CTD, connective tissue disease; HP, hypersensitivity pneumonitis*.

**Table 2:** Relative frequency of interstitial lung disease subtypes.

***N* (%)**		**Source/Case Ascertainment**	**Time Period**	**IPF**	**CTD**	**Sarcoid**	**HP**	**Drug**	**Occupational**	**Unclassifiable**	**Other**
**North America**											
New Mexico, USA	202 (incident cases)	County Chart Review	1988–1990	63 (31.2)	18 (8.9)	16 (7.9)	3 (1.5)	7 (3.5)	21 (10.4)	20 (9.9)	54 (26.7)
New Mexico, USA	258 (prevalent cases)	County Chart Review	1988–1990	58 (22.5)	33 (12.8)	30 (11.6)	–	5 (1.9)	36 (14.0)	29 (11.2)	67 (26.0)
Quebec, Canada	52	Indigenous Population MDD	2006–2013	27 (51.9)	6 (11.5)	1 (1.9)	1 (1.9)	–	1 (1.9)	3 (5.8)	13 (25.0)
Canada	1,285	Multi Center MDD	2016–2017	317 (24.7)	428 (33.3)	41 (3.2)	97 (7.5)	–	–	286 (22.3)	116 (9.0)
**Europe**											
Flanders (Belgium)	264 (incident cases)	Multi Center Survey	1992–1996	59 (22.3)	19 (7.2)	69 (26.1)	32 (12.1)	12 (4.5)	18 (6.8)	27 (10.2)	28 (10.6)
Flanders (Belgium)	362 (prevalent cases)	Multi Center Survey	1992–1996	72 (20.0)	27 (7.5)	112 (30.9)	47 (13.0)	12 (3.3)	20 (5.5)	33 (9.1)	39 (10.8)
Greece	259 (incident cases)	Multi Center Survey	2004	52 (20.1)	30 (11.6)	60 (23.2)	7 (2.7)	4 (1.5)	8 (3.1)	40 (15.4)	58 (22.4)
Greece	967 (prevalent cases)	Multi Center Survey	2004	189 (19.5)	120 (12.4)	330 (34.1)	25 (2.6)	17 (1.8)	20 (2.0)	82 (8.5)	184 (19.0)
Denmark	431 (incident cases)	Single Center MDD	2003–2009	121 (28.1)	54 (12.5)	–	32 (7.4)	20 (4.6)	–	62 (14.4)	142 (32.9)
Spain	511 (incident cases)	Multi Center Survey	2000–2001	197 (38.6)	51 (10.0)	76 (14.9)	34 (6.6)	17 (3.3)	–	26 (5.1)	110 (21.5)
Italy	3,152	Multi Center Survey	1998–2005	864 (27.4)	–	1,063 (33.7)	93 (2.9)	39 (1.2)	–	–	–
Paris, France	848 (prevalent cases)	County MDD	2012	98 (11.5)	145 (17.1)	361 (42.6)	28 (3.3)	31 (3.7)	42 (5.0)	66 (7.8)	77 (9.1)
**Asia**											
Turkey	2,245 (incident cases)	Multi Center Survey	2007–2009	408 (18.2)	201 (9.0)	771 (34.3)	82 (3.7)	35 (1.6)	241 (10.7)	99 (4.4)	408 (18.2)
India	566 (incident cases)	Single Center MDD	2015–2017	130 (23.0)	77 (13.6)	217 (38.3)	69 (12.2)	5 (0.9)	6 (1.1)	–	62 (11.0)
India	803 (prevalent cases)	Single Center MDD	2015–2017	170 (21.2)	102 (12.7)	339 (42.2)	86 (10.7)	6 (0.7)	7 (0.9)	7 (0.9)	86 (10.7)
India	1,084 (incident cases)	Multi Center MDD	2012–2015	148 (13.7)	151 (13.9)	85 (7.8)	513 (47.3)	3 (0.3)	33 (3.0)	2 (0.2)	149 (13.7)
Pakistan	253	Single Center Chart Review	2016–2018	95 (37.5)	23 (9.1)	11 (4.3)	31 (12.3)	–	3 (1.2)	4 (1.6)	86 (34.0)
China (Guangzhou)	1,945 (incident cases)	Single Center MDD	2012–2017	395 (20.3)	356 (18.3)	123 (6.3)	59 (3.0)	13 (0.7)	13 (0.7)	285 (14.7)	701 (36.0)
China (Beijing)	2,615 (incident cases)	Single Center Chart Review	2000–2012	692 (26.5)	631 (24.1)	147 (5.6)	62 (2.4)	28 (1.1)	58 (2.2)	344 (13.2)	653 (25.0)
**Other**											
Saudi Arabia	330 (incident cases)	Single Center MDD	2008–2011	77 (23.3)	115 (34.8)	67 (20)	21 (6.3)	4 (1.2)	–	6 (1.8)	40 (12.1)
Australia	705	Multi Center Survey	2016–2019	240 (34.0)	125 (17.7)	44 (6.2)	66 (9.4)	7 (1.0)	11 (1.6)	51 (7.2)	161 (22.8)

*MDD, multidisciplinary discussion; ILD, interstitial lung disease; IPF, idiopathic pulmonary fibrosis; CTD, connective tissue disease; HP, hypersensitivity pneumonitis*.

More recently, a Canadian epidemiologic study evaluated the distribution of ILD subtypes among the indigenous population living in Northern Quebec between 2006 and 2013 (6). Patients were identified using a combination of hospitalization databases, home oxygen use registries and physician surveys. Individual cases were adjudicated *via* multidisciplinary discussion (MDD) and a total of 52 cases were identified as definite ILD. There was a high prevalence of IPF (52%) in the cohort followed by CTD-ILD (11.5%). There was a much lower prevalence of occupational lung disease (1.9%) and sarcoidosis (1.9%) than had been observed in Bernalillo County, likely due to different characteristics and risk factors of the underlying source population.

In contrast to the Bernalillo County and Northern Quebec, which were population-based studies, the Canadian Registry for Pulmonary Fibrosis (CARE-PF), a multi-center, prospective registry that recruited patients from six specialized Canadian ILD clinics between 2016 and 2017, noted a much higher frequency of CTD-ILD (33.3%) followed by IPF (24.7%) and unclassifiable ILD (22.3%) (7). All cases were adjudicated *via* MDD. The mean age of the ILD cohort was 64.8 years with a slightly higher preponderance of females (50.7%). The authors hypothesized that the higher proportion of unclassifiable ILD in their cohort was due to a combination of factors including the complexity of cases seen at tertiary care referral centers and the utilization of strict diagnostic criteria for IPF, chronic hypersensitivity pneumonitis (HP), and idiopathic non-specific interstitial pneumonia (NSIP), the latter of which required biopsy confirmation. Thus, it is possible that the prevalence of IPF, HP and NSIP were under estimated in this cohort because of the diagnostic criteria applied.

### Europe

Perhaps the most robust epidemiological data examining comparative frequencies of ILDs comes from national registry studies conducted across Europe, the majority of which have demonstrated a high prevalence of IPF and sarcoidosis.

One of the first prospective registry studies evaluated the epidemiology of ILD in Flanders, the northern region of Belgium, between 1992 and 1996 (8). A total of 362 patients were recruited from 20 centers across 5 provinces *via* enrollment surveys completed by physicians. The mean age of the ILD cohort was 52 years old. There was a high prevalence of sarcoidosis (31% when stage I was included, 22% when stage I was excluded), followed by IPF (20%), HP (13%), and CTD-ILD (7.5%). Approximately 9.1% of cases were unclassifiable. Notably, the male to female ratio was variable across disease processes with pneumoconiosis and IPF more prevalent among men (M/F ratio of 2.3 and 1.4, respectively) while CTD-ILD was more common in women (M/F ratio of 0.8). Of the HP cases, the majority (75%) were associated with pigeon breeding, impacting more men than women (M/F ratio of 1.5).

A similar distribution of ILD subtypes was observed in Greece (9). In a multi-center ILD registry study, 967 patients were recruited from pulmonary divisions across the country. There was a slightly higher proportion of females in the cohort (53.6%). The mean age of the male population was 58 years old, and the mean age of the female population was 59.3 years old. Sarcoidosis was the most commonly observed ILD subtype (34.1%), followed by IPF (19.5%) and CTD-ILD (12.4%). The prevalence of HP was relatively low (2.6%) and unclassifiable ILDs comprised 8.5% of the cohort. The Greek cohort, similar to other European studies, included stage I sarcoidosis (isolated hilar adenopathy), which may have contributed to the higher proportion of sarcoid cases relative to North American cohorts, which generally only included sarcoidosis stages II–IV (stage II: hilar adenopathy with parenchymal involvement, stage III: parenchymal involvement without lymphadenopathy, and stage IV: predominantly fibrotic disease) in their registries.

A Danish study that sought to describe the incidence of ILDs in central Denmark recruited 431 patients from a single center between 2003 and 2009 (10). Cases were adjudicated *via* MDD. The mean age of the cohort was 61 years and 55% were male. The overall incidence of ILD was 4.1 cases per 100,000 person-years. The study reported a rising annual incidence rate with a peak of 6.6 cases per 100,000 person-years in 2009. The most common ILD was IPF (28%), followed by CTD-ILD (12.5%) and HP (7%). IPF and HP was more common in men (77% and 63%, respectively), while CTD-ILD was more common among women (59%). Notably, sarcoidosis was not included in this cohort.

In Spain, a multicenter registry study that enrolled patients *via* surveys completed by 23 pulmonary medicine clinics between 2000 and 2001 noted an estimated ILD incidence of 7.6 per 100,000 person-years (11). IPF was the most common ILD subtype (38.6%), followed by sarcoidosis (14.9%), CTD-ILD (10%) and HP (6.6%). Approximately 5% of the cases were unclassifiable. Among the CTD-ILD cohort, rheumatoid arthritis was the most common etiology. Similar to observations from the Belgium cohort, pigeon breeding was the most common exposure associated with a diagnosis of HP.

In Italy, the Registro Italiano Pneumopatic Infiltrative Diffuse (RIPID) enrolled 3,152 patients *via* surveys completed by 79 centers across 20 regions (12). The mean age at diagnosis was 54 years with a slightly higher proportion of females (50.9%) in the registry. Sarcoidosis was the most frequently reported ILD (33.7%), followed by IPF (27.4%), which together represented more than 60% of cases. 93 cases (2.9%) of HP were identified.

More recent epidemiologic studies in Europe have focused on using large databases (healthcare claims, mortality, social security) as an alternative to hospital-based registries to define the population burden of ILD. In France, a study that described the population burden of chronic ILDs among people living in Seine-Saint-Denis, a multi-ethnic urbanized area of Greater Paris, noted much higher ILD point prevalence rates than prior registry-based studies (13). Patients were recruited from both physicians' offices and the social security system between January and December 2012. A total of 848 cases were reviewed and validated centrally by an expert MDD. The median age was 55.7 years old with an equal distribution of males and females. The overall incidence of ILD was 18.3 per 100,000 person-years and prevalence was 71 per 100,000 people. In contrast to other European studies, the prevalence of IPF was much lower in this cohort. The most common diagnosis was sarcoidosis (42.6%), followed by CTD-ILD (17.1%), IPF (11.5%) and occupational lung disease (5%). There was a low prevalence of HP (3.3%). The ancestry-standardized prevalence rates noted a higher frequency of sarcoidosis and CTD-ILDs among patients from North Africa (60 and 26.9 per 100,000, respectively) than in Europeans (10.7 and 5.7 per 100,000, respectively). The ancestry-standardized prevalence of IPF was higher among North Africans than Europeans and Afro-Caribbean (26.9, 5.8, and 4.2 per 100,000, respectively). Adjusted multivariable models demonstrated increased risk of sarcoidosis in Afro-Caribbean (OR 2.9) and North Africans (OR 1.9). The risk of CTD-ILDs was also increased in Afro-Caribbean (OR 4.4) relative to their European counterparts. The authors noted that the area of Seine-Saint-Denis is demographically distinct from that of the general French population with a younger mean age and a higher proportion of people of extra-European ancestry and thus may not be generalizable to the French population at-large. The low prevalence of IPF is likely related to the age distribution, which was skewed toward younger patients.

### Asia

Compared to Europe and North America, the English language literature on ILD in Asia has until recently been quite limited. In the last few years, several epidemiologic studies evaluating relative frequency of ILDs have emerged from Turkey, India, Pakistan and China.

In a multicenter cohort study involving recruitment from 31 centers in Turkey, a total of 2,245 cases were identified of which 48.2% were males and 51.8% were females. The mean age was 52 years old. The overall incidence of ILD was 25.8 per 100,000 (14). Sarcoidosis was the most common ILD subtype (34.3%) followed by IPF (18.2%), occupational lung disease (10.7%) and CTD-ILD (9%). There was a low prevalence of HP (3.7%) in the cohort. The study also subcategorized disease burden by sex and age. Among females, sarcoid was the most prevalent (53%), followed by an equal distribution of CTD-ILD (15%) and IPF (15%). For men, the proportion of patients with sarcoid, pneumoconiosis and IPF was nearly equivalent (25% sarcoid, 25% IPF, 24% pneumoconiosis) while prevalence of CTD-ILD (6%) was notably lower. With age, the distributions shifted. For men over the age of 50, IPF was the most common ILD (45%) followed by pneumoconiosis (13%) and then sarcoidosis (8%). For men under 50, sarcoidosis was the most prevalent (42%), followed by pneumoconiosis (36%) with a relatively low prevalence of IPF (6%). High rates of pneumoconiosis in Turkey were postulated to be linked to the denim sandblasting profession resulting in a high burden of silicosis among those with occupational lung diseases.

A few large database studies have evaluated the epidemiology of ILD in India. One single center study recruited 803 patients between 2015 and 2017 and adjudicated cases *via* MDD (15). The mean age of the cohort was 50.6 years old with 50.2% women. Sarcoidosis (42.2%) and IPF (21.2%) were the most common ILD subtypes followed by CTD-ILD (12.7%) and HP (10.7%). Most sarcoid patients (63.4%) had stage II or III disease. RA and systemic sclerosis were the most commonly identified CTD-ILD. Of the patients with HP, the most common exposure was farming (59.3%), followed by exposure to bird feathers (15.1%).

The second epidemiological evaluation of ILD frequencies in India involved a multi-center cohort study, which recruited 1,084 patients from 27 centers between 2012 and 2015 (16). Cases were adjudicated *via* a central MDD. The mean age of registry participants was 55.3 years and 47.2% were male. HP was the final diagnosis in a majority of cases (47.3%), followed by CTD-ILD (13.9%), IPF (13.7%), sarcoidosis (7.8%), and pneumoconiosis (3%). Among patients with HP, 48.1% had been exposed to air coolers, 26.3% to air conditioners, 21.4% to birds and 20.7% to mold in their homes. RA was the most common type of CTD-ILD (38.4%) followed by scleroderma (22.5%). Silicosis was the most common occupational lung disease. The authors noted that compared to other epidemiological studies, a smaller proportion (7.5%) of patients had undergone lung biopsy, which may have led to an underestimation of IPF prevalence, especially as histopathology is often used to differentiate fibrotic HP form IPF. Although the data was presented in aggregate, there was significant within country variability in geographic prevalence of ILD subtypes.

In Pakistan, 253 patients were identified *via* chart review from a single center in Karachi between 2016 and 2018 (17). There was a clear predominance of females (69%) in the registry and the mean age was 49 years old. IPF was the most common disease subtype (37.5%) followed by HP (12.3%), CTD-ILD (9.1%) and sarcoidosis (4.3%). Approximately 37% of patients reported exposure to birds including parakeets, parrots, hens and pigeons.

Two studies examined the epidemiology of ILD in China. The first, retrospectively identified 1,945 patients seen in Guangzhou Institute of Respiratory Health (Southern China) between 2012 and 2017 (18). Case adjudication was done *via* MDD. The mean age at time of diagnosis was 57.9 years and 55.5% of patients were male. The most common ILD subtype was IPF (20.3%), followed by CTD-ILD (18.3%) and interstitial pneumonia with autoimmune features (IPAF) (17.9%). Among the CTD-ILD subgroup, there was a higher proportion of females (60.1%), and RA (32.6%), myositis (25%) and primary Sjogren disease (14%) were the most common CTD subtypes. Although other studies had reported a high percentage of RA-ILD among their CTD-ILD cohorts, the Guangzhou Institute had a much higher prevalence of myositis-ILD than what had been observed in North America, Europe or other parts of Asia. Only 3% of patients were diagnosed with HP. The most common environmental exposure was mold/mildew followed by farming and bird exposure. Relative to other cohorts, especially in Asia, a large number of patients underwent lung biopsy (42.1%).

A second study from China evaluated the distribution of ILD among 2,615 patients of Chinese ancestry admitted to a hospital in Beijing between 2000 and 2012. Patients were identified through chart review. The mean age at diagnosis was 61 years and 59.3% of the cohort was female (19). IPF was the most common ILD subtype (26.5%), followed by CTD-ILD (24.1%) and unclassifiable IIP (13.2%). The most common types of CTD-ILD were Sjogren disease (11.2%) and RA-ILD (4.6%). Sarcoidosis accounted for 5.6% of cases and pneumoconiosis accounted for 2.2%.

### Middle East

There is limited literature on the epidemiology of ILD in the Middle East. One study examined the frequency of ILD subtypes in Saudi Arabia by prospectively recruiting patients with new ILD diagnoses from a single tertiary care center between 2008 and 2011 (20). Cases were adjudicated *via* MDD. A total of 330 patients of native Saudi origin were enrolled with a mean age of 55.4 years and a predominance of females (61.2%) in the cohort. CTD-ILD (34.8%) was the most commonly diagnosed ILD, which included patients diagnosed with IPAF, followed by IPF (23.3%), sarcoidosis (20%), and HP (6.3%). The distribution of sarcoidosis ranged from 12% in stage I, 31% in stage II, 6% in stage III, to 51% in stage IV. The authors postulated that the higher proportion of stage IV sarcoid cases was in part due to referral bias as many patients with stage I and II disease were likely managed in the community. Among patients with HP, an exposure was identified in 66.7% of cases with the most common being birds. Surgical lung biopsies were performed in 22.7% of cases.

### Australia

The Australian Interstitial Lung Disease Registry (AILDR) is the largest longitudinal cohort study of ILD in Australia and New Zealand (21). A total of 1,061 patients were recruited from four ILD centers across the continent between 2016 and 2019 *via* surveys distributed to physicians. The mean age of participants was 68.3 years with 54.7% male. The most common diagnosis was IPF (34%) followed by CTD-ILD (17.7%), HP (9.4%) and sarcoidosis (6.2%). The registry also included cases of IPAF (0.4%), which was significantly lower than the frequency of IPAF cases observed in China and the Middle East.

## Global Trends in Interstitial Lung Disease Mortality

The Global Burden of Disease Study noted that ILDs contributed to 0.26% of all-cause mortality in 2017 and that there had been an 86% increase in ILD-related years of life lost over the past two decades (22). The 5-year survival among patients with ILD has been estimated to be 56% (23). However, there is significant heterogeneity in survival by ILD subtypes. The 5-year survival in a national cohort of Danish patients was 34% among those with IPF, 74% in patients with idiopathic NSIP, and 93% among patients with HP (10). Given this variability, current literature has primarily focused on evaluating global trends in ILD mortality by subtype, with most studies focused on IPF.

IPF is a progressive fibrotic lung disease associated with insidious decline in lung function. Historically, the median survival of IPF has been estimated to range from 2 to 5 years (24, 25). However, there is significant variability by subgroup with longer median survival times among younger patients (26). More recent data suggests that in addition to age-related variability in IPF survival, there may be geographic variability as well. In a review of IPF mortality across 10 countries between 1999 and 2012, the age standardized mortality ranged from 4 to 10 per 100,000 with the lowest mortality rates observed in Sweden, Spain, and New Zealand and the highest mortality rates observed in the United Kingdom and Japan (27). Within the United States, approximately 0.7% of all deaths that occurred between 2004 and 2016 had a diagnosis of pulmonary fibrosis and mortality rates were lower among women, Black, and Asians. There was significant variability in survival by state (28). The reasons for this variability in outcomes both within countries and between countries is unclear. Notably, the majority of these studies were conducted prior to approval and widespread adoption of antifibrotic therapies (pirfenidone and nintedanib), which have been shown to slow disease progression and improve survival. Thus, newer studies may demonstrate changing disease trajectories.

More recently, there has been increasing interest in understanding the prognosis of patients with non-IPF progressive fibrosing interstitial lung disease (PF-ILD) in light of clinical data suggesting that these patients may also benefit from antifibrotic therapies (29). In France, the median overall survival for patients with non-IPF PF-ILD was 3.7 years. Among this subgroup, patients with sarcoidosis had the longest median survival time (7.9 years) and patients with non-HP exposure related ILD had the shortest (2.4 years). These findings are consistent with prior literature that has suggested that the prognosis for patients with sarcoidosis may be better than other forms of ILD.

## Discussion

There are limited epidemiologic studies describing the global burden and relatively geographic heterogeneity of interstitial lung disease subtypes, and there are continents (e.g., South America and Africa) without English language literature on the topic. We found that among seventeen methodologically heterogenous studies that examined the incidence, prevalence and relative frequencies of ILD subtypes, the incidence of ILD ranged from 1 to 31.5 per 100,000 person-years and prevalence ranged from 6.3 to 71 per 100,000 people (Table 1). In North America and Europe, IPF and sarcoidosis were generally the most prevalent ILDs with the prevalence of IPF ranging from 1.3 per 100,000 in Belgium to 20.2 per 100,000 among males in Bernalillo County, New Mexico. The prevalence of sarcoidosis ranged from 1.94 per 100,000 in Belgium to 30.2 per 100,000 in Paris, France. The relative frequency of occupational interstitial lung disease was highest among patients in Bernalillo County (14%) and Turkey (10.7%) (Table 2, Figure 1). The relative frequency of HP was higher in Asia, particularly in India (10.7–47.3%) and Pakistan (12.3%), compared to most of the North American and European cohorts. The relative frequency of CTD-ILD demonstrated the greatest geographic variability, ranging from 7.5% of cases in Belgium to 33.3% of cases in Canada and 34.8% of cases in Saudi Arabia.

The reasons for this geographic heterogeneity is likely due to combination of methodological factors and variability in characteristics of the underlying source populations. Most registry-based epidemiologic studies have historically relied on individual patient recruitment from pulmonary clinics, which can lead to selection bias of the referral base, underestimation of true disease burden, and may not be representative of the general ILD population. This type of recruitment is also more likely to exclude certain types of ILDs like sarcoidosis and CTD-ILD, which may be managed by internal medicine physicians or rheumatologists. The Danish cohort excluded sarcoidosis from its registry for this reason (10).

Changing definitions of ILD subtypes due to evolving society guidelines also pose methodological challenges in quantifying temporal trends and comparing changes in relative frequency of ILDs over time. This is particularly true for idiopathic interstitial pneumonias, specifically IPF, for which there have been multiple iterations of clinical practice guidelines over the last decade (30–32). Additionally, new guidelines describing the entity of interstitial pneumonia with autoimmune features (IPAF) have led newer registries to qualify IPAF as a distinct ILD subtype, while other have collated IPAF under the broader umbrella term idiopathic interstitial pneumonia or alternatively under CTD-ILD itself (18, 20, 21, 33). This may partially explain the geographic variability in frequency of CTD-ILD noted in the literature.

Variable methods for case adjudication and differences in diagnostic confidence thresholds likely also contributed to the geographic heterogeneity noted. Of the 17 studies reviewed, approximately half explicitly reported MDD as a requirement for case adjudication. The remainder, primarily multicenter national registries, relied on enrollment surveys completed by referring physicians. Although these surveys included details about patient demographics, pulmonary function tests, high resolution CT scans and pathology when available, the studies did not uniformly report whether MDD was required prior to a final ILD diagnosis. In addition, as there are no universally agreed upon thresholds for diagnostic confidence, some variability may be explained by the stringency of diagnostic criteria applied. For example, registries like the Canadian national registry, which applied more stringent criteria that required biopsy confirmation for a diagnosis of idiopathic NSIP, may have underestimated the prevalence of some ILDs and had a higher proportion of unclassifiable cases (7). On the other hand, very few cases in the Indian registries had pathology available (16). Biopsies are often used to differentiate HP from IPF. Using history and radiology alone in these registries may have led to higher prevalence of HP in those cohorts.

Despite these methodological limitations, some differences observed between registry-based studies, may represent true differences in the demographics and exposures of the source populations. For example, in the Parisian cohort, which specifically evaluated the epidemiology of ILD among Seine-Saint-Denis, a multi-ethnic county of Greater Paris, the calculated ancestry-standardized incidence and prevalence rates of sarcoidosis and CTD-ILDs were higher among patients of North African descent (13). In India, the high prevalence of HP was partially attributed to widespread use of evaporative air coolers, which are prone to mold growth (16). Cohorts with predominantly younger patients or a higher proportion of women noted higher rates of CTD-ILD and lower rates of IPF. In Turkey and Belgium, the sex-standardized frequency of ILD subtypes favored CTD-ILD among women and pneumoconiosis among men (8, 14). A more complete understanding of these risk factors and the role that genetic ancestry may play in ILD risk can lead to important insight into predisposing factors that contribute to both ILD development and progression. Identification of ILD clusters can shed light on new exposures, their pathogenic mechanisms, and create an opportunity to intervene on modifiable occupation and environmental risk factors.

Mortality data examining the geographic variability in survival by ILD subtype is limited. Current literature suggests that IPF has the worst prognosis. Cohorts with a high proportion of patients with IPF may note higher overall ILD mortality rates associated with high healthcare utilization rates. IPF specific mortality rates may vary by geography. Whether this is due to underlying demographics of source populations or reflective of access to healthcare resources is unclear. Better understanding the reasons for geographic variability in ILD outcomes by subtype can expand our current clinical understanding of disease as well as identify care gaps for potential targeted intervention.

## Areas for Improvement and Future Directions

There are three areas where we feel additional work is needed to better understand the global burden of interstitial lung diseases. First, a standard ontology with diagnostic confidence thresholds is needed for comparative epidemiology studies of ILD (34). As demonstrated by this review, different authors choose different categorizations schema, employ variable diagnostic thresholds, and utilize different methodologies for establishing diagnosis. A unified set of diagnostic categories and criteria for this work would greatly help aggregate studies into informative reviews.

Second, more globally representative data should be published in English language journals or alternatively be translated into English and made available through open access. Most available epidemiologic studies in English have focused on evaluating disease burden in North America and Europe with only recent data from Asia. There are thus significant knowledge gaps regarding frequency of ILD subtypes in South America and Africa. Japan and South Korea, both major centers for ILD research, are also underrepresented in the English language literature.

Some knowledge gaps may also be due to healthcare infrastructure challenges in developing countries, particularly in South America and Africa, where access to tertiary care referral centers with dedicated chest radiologists and pulmonologists specializing in the diagnosis and management of ILD is limited. In addition, the paucity of data from many developing countries may reflect competing public health priorities, particularly of pulmonary diseases like tuberculosis, which disproportionately impact Asia, South America, and Africa. Multinational collaborative registries between ILD referral centers, like the recently established Latin American Idiopathic Pulmonary Fibrosis Registry (REFIPI), have the potential to consolidate resources and bridge this knowledge gap (35). Building on these types of registries to better understand the burden and relative frequencies of ILD in understudied countries would be informative, especially in light of increasing literature exploring the complex interplay between genetics, environment and ILD.

Third, the inclusion of larger and more community-based cohorts is needed. Extrapolating regional or national epidemiology from single-center, specialty-based cohorts is likely leading to significant mischaracterization of the true distribution of ILDs. The Bernalillo County, New Mexico registry was among the first to use International Classification of Disease (ICD) codes followed by chart review in an attempt to provide more representative and inclusive data, and this may in part explain the higher incidence and prevalence reported (5). The electronic health record (EHR) is a potentially powerful tool for epidemiologists to address the issue of inclusion and generalizability. To date, most EHR based studies in ILD have focused on describing the epidemiology of individual ILD entities, most commonly IPF (26, 36), rather than evaluating comparative frequencies. One study that explored the epidemiology of IPF in U.S. Medicare claims data reported an annual IPF incidence of 93.7 cases per 100,000 person-years and a cumulative prevalence of 494.5 cases per 100,000 people in 2011 (26). These estimates are much higher than incidence and prevalence estimates noted in the majority of registry-based studies. It is possible that the higher incidence and prevalence noted in EHR-based studies reflects overdiagnosis in the absence of multidisciplinary case validation. Alternatively, it is possible that registry-based studies, many of whom recruit from tertiary care referral centers, underestimate population burden of disease. Future work that can leverage claims data as a screening tool to identify possible ILD cases with additional case validation to verify the accuracy of claims-based algorithms may facilitate more accurate estimates of ILD epidemiology. EHR data could also create an opportunity to recruit patients into national registries by leveraging electronic alerts to encourage referral to subspecialty centers for patients who meet EHR screening criteria for ILD.

Improving the functionality of EHR data for research purposes will require a concerted effort by the broader ILD community. Historically, ICD codes have been the primary means of EHR disease identification. However, ICD codes were developed for billing purposes with less attention given to specificity of diagnosis. This has limited their effectiveness for use in research studies. A concerted effort to adopt standardized codes with an emphasis on diagnostic accuracy has the potential to drastically expand the efficiency and speed with which we are able to draw from large, demographically and clinically diverse population-based cohorts. The opportunity to link EHR data with mortality data as is already done the United States Veterans Affairs Healthcare System, can further accelerate our progress.

We believe the ILD research community should organize a global summit to define a shared ontology for disease classification, set diagnostic confidence thresholds, and commit to conducting global claims and EHR-based epidemiologic studies in a standardized fashion. These data could be published in a shared issue of the major specialty journals. Aggregating and sharing data would provide a unique opportunity for international collaboration as our understanding of ILD continues to grow and evolve. These large, community-based longitudinal cohorts would also allow for tracking of global trends and be a valuable resource for collective study.

## Conclusions

In conclusion, we have summarized the English language literature of the comparative epidemiology of ILD and demonstrated that there is significant geographic heterogeneity in the global disease burden and outcomes. These differences may represent true differences based on demographics and exposures of the source populations or methodological differences in patient recruitment (registry vs. population-based cohorts) and disease classification. Better understanding the geographic and temporal patterns of disease prevalence and identifying clusters of ILD subtypes can facilitate improved understanding of emerging risk factors and help identify targets for intervention. Future work, including a standardized ontology for classification, more globally inclusive studies, and leveraging EHR data with uniform coding practices to develop more generalizable, community-based cohorts, will help advance our understanding of this important group of diseases. We encourage the international ILD community to organize and address this unmet need.

## Author Contributions

BK, VC, HC, and CV contributed to conception and design of the review. BK wrote the first draft of the manuscript. VC, HC, and CV provided critical feedback. All authors contributed to manuscript revision, read, and approved the submitted version.

## Funding

Research reported in this publication was supported by the National Heart Lung and Blood Institute of the NIH under Awards K12HL138046 and K24HL12131.

## Author Disclaimer

The content is solely the responsibility of the authors and do not necessarily represent the official views of the NIH.

## Conflict of Interest

The authors declare that the research was conducted in the absence of any commercial or financial relationships that could be construed as a potential conflict of interest.

## Publisher's Note

All claims expressed in this article are solely those of the authors and do not necessarily represent those of their affiliated organizations, or those of the publisher, the editors and the reviewers. Any product that may be evaluated in this article, or claim that may be made by its manufacturer, is not guaranteed or endorsed by the publisher.
